# Supplementary material for: Intermittent Fasting Alleviates Risk Markers in a Murine Model of Ulcerative Colitis by Modulating the Gut Microbiome and Metabolome
Source: Nutrients. 2022 Dec 14;14(24):5311. doi: 10.3390/nu14245311 (PMC9788567; doi:10.3390/nu14245311)
Supplement: Supplementary file 1 [file nutrients-14-05311-s001.zip › nutrients-2007400-supplementary.pdf]

A

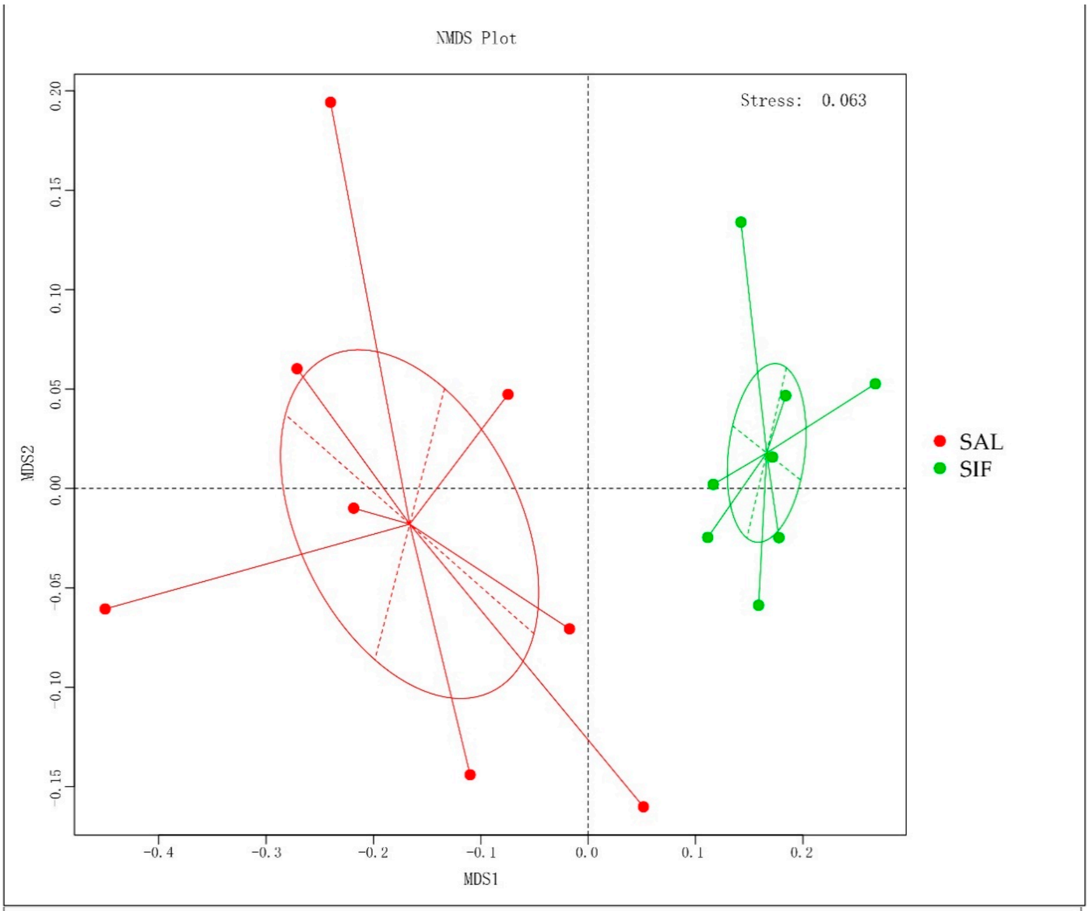

NMDS Plot
Stress: 0.063
MDS1
MDS2
−0.4
−0.3
−0.2
−0.1
0.0
0.1
0.2
−0.15
−0.10
−0.05
0.00
0.05
0.10
0.15
0.20
SAL
SIF

B

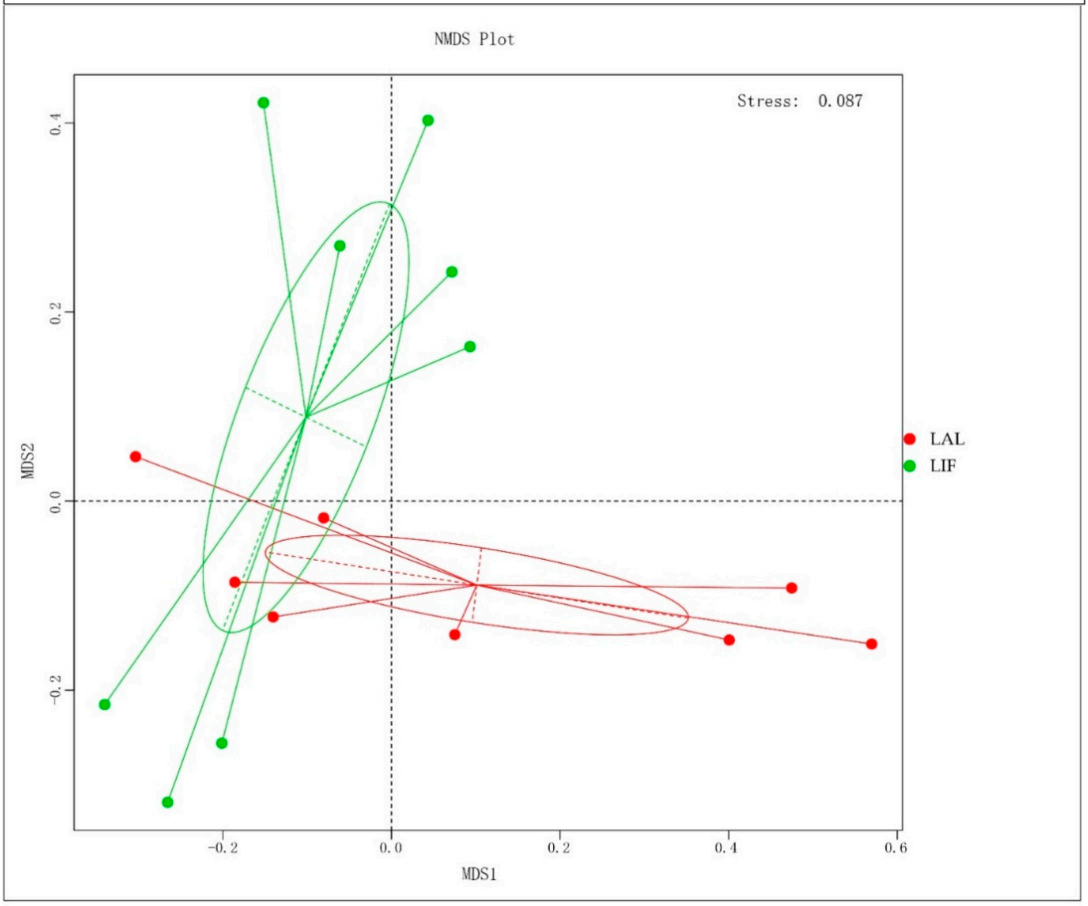

NMDS Plot
Stress: 0.087
MDS1
MDS2
−0.2
0.0
0.2
0.4
0.6
−0.2
0.0
0.2
0.4
LAL
LIF

Supplementary Materials Figure S1. IF impacts gut microbiota. **A**, NMDS analysis of gut microbiota with short-term IF; **B**, NMDS analysis of gut microbiota with long-term IF. SAL: Short-term of ad libitum-fed group; SIF: Short-term of intermittent fasting group; LAL: Long-term of ad libitum-fed group; LIF: Long-term of intermittent fasting group.

A

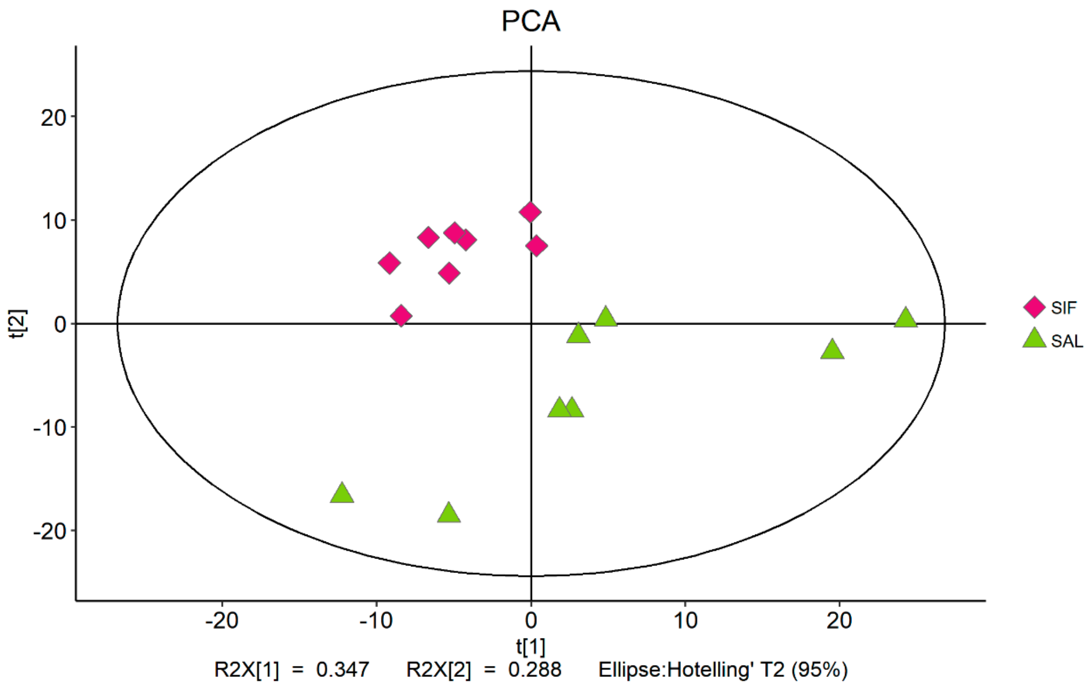

B

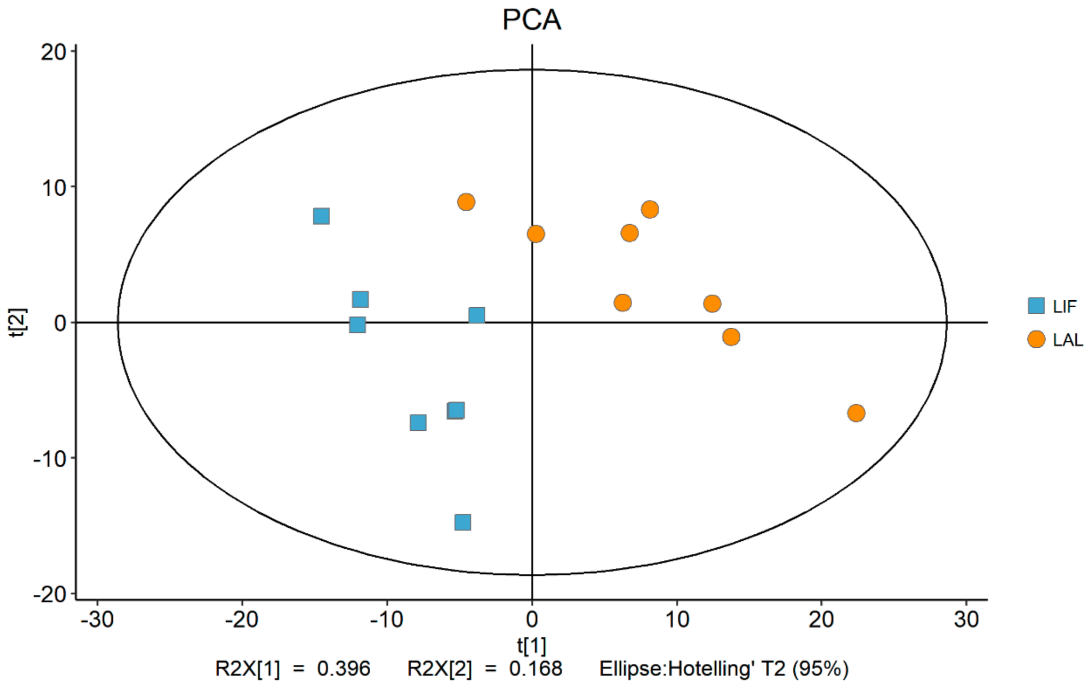

C

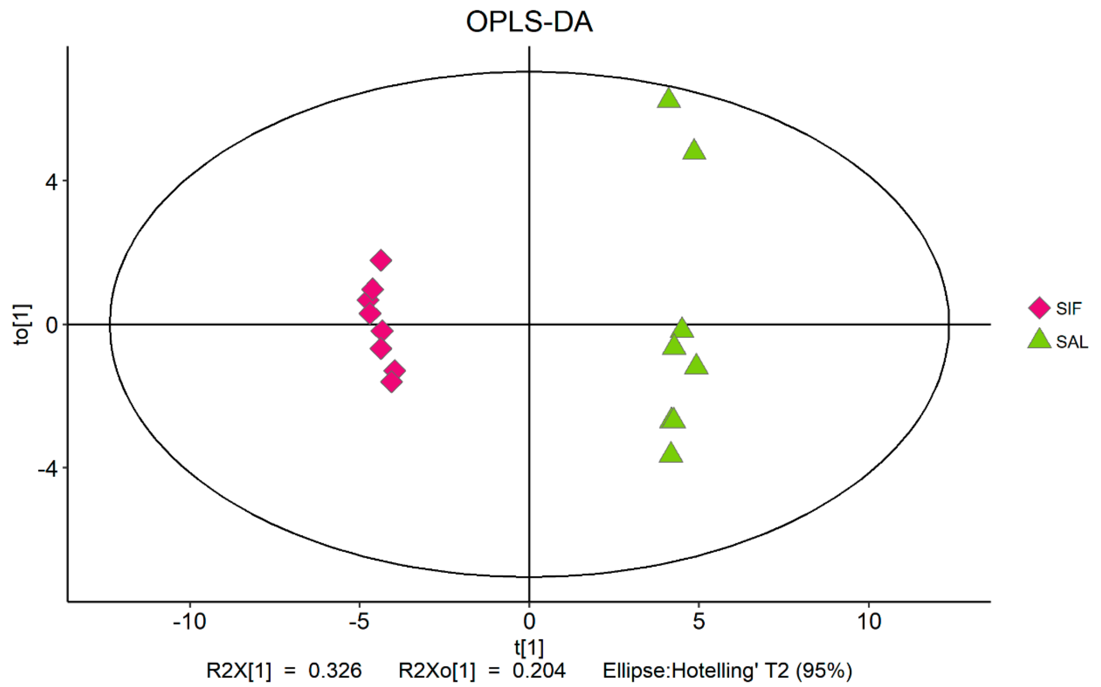
OPLS-DA
to[1]
4
0
-4
-10
-5
0
5
10
t[1]
R2X[1] = 0.326 R2Xo[1] = 0.204 Ellipse:Hotelling' T2 (95%)
SIF
SAL

D

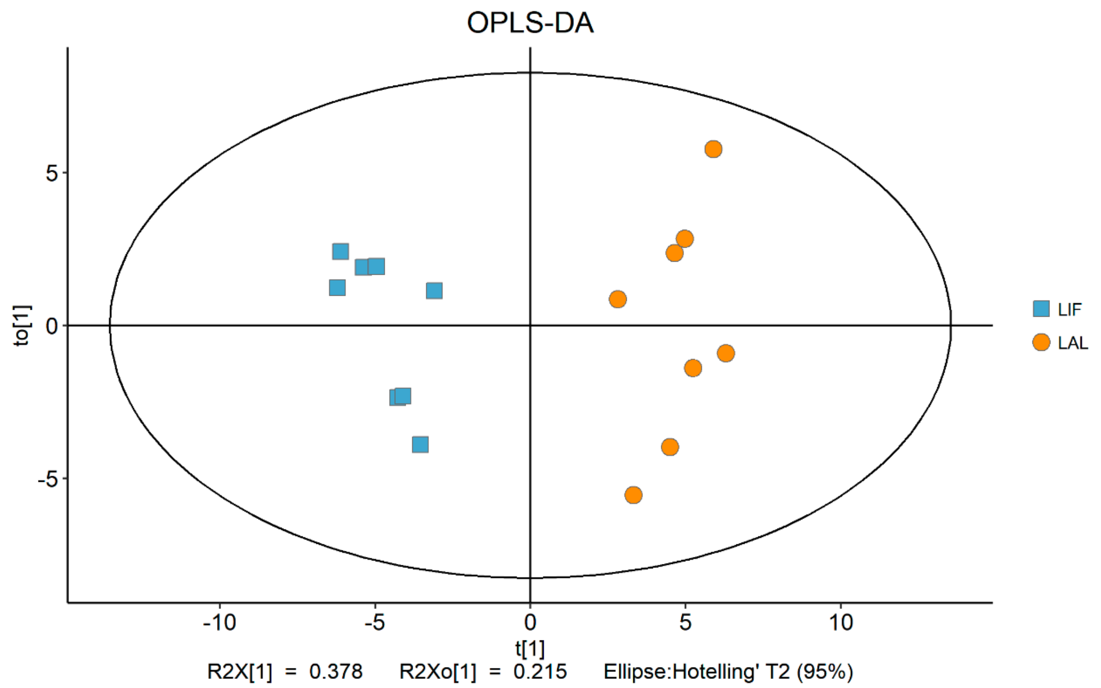
OPLS-DA
to[1]
5
0
-5
-10
-5
0
5
10
t[1]
R2X[1] = 0.378 R2Xo[1] = 0.215 Ellipse:Hotelling' T2 (95%)
LIF
LAL

E

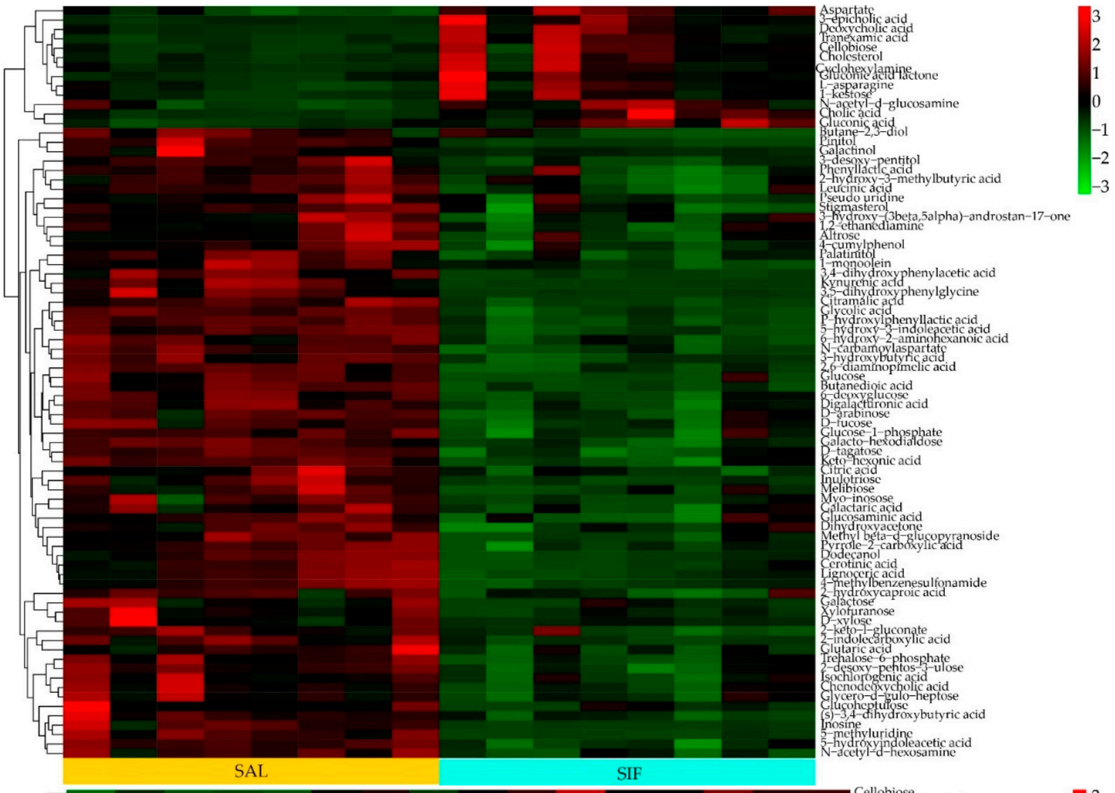

F

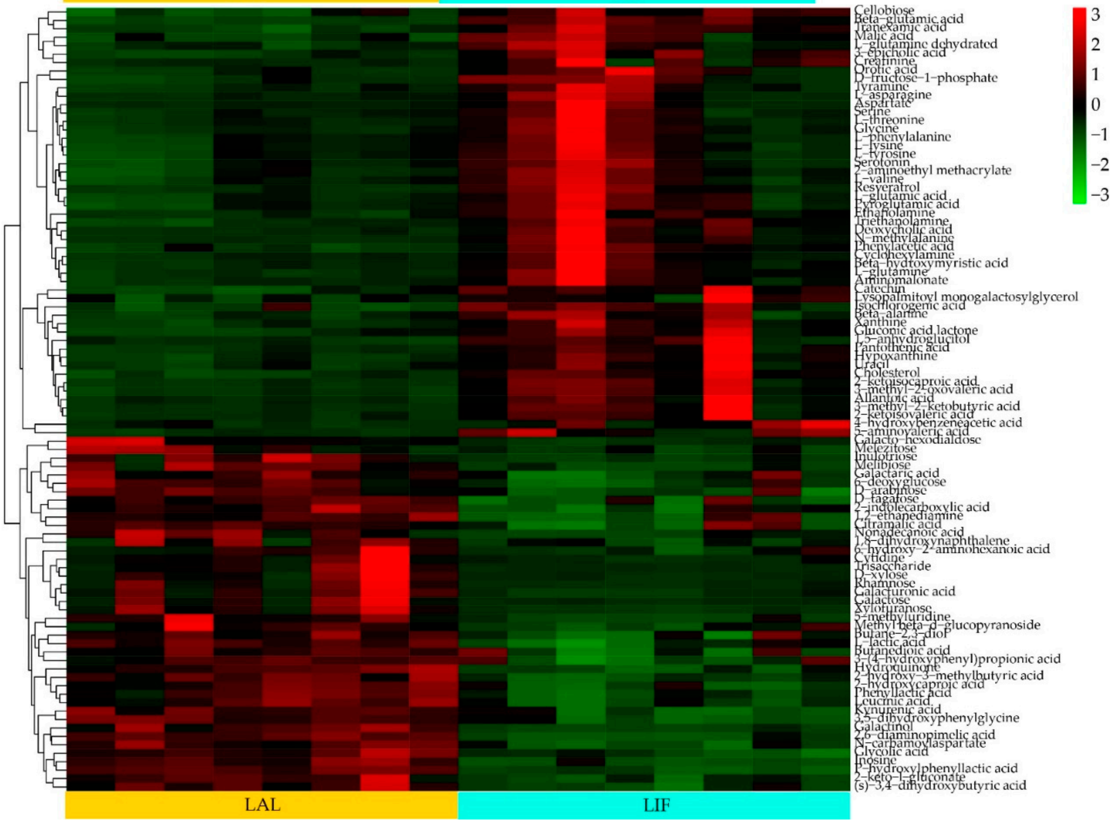

Supplementary Materials Figure S2. Metabolomic profiles of fecal after IF**. A**, PCA analysis in SAL and SIF groups; **B**, PCA analysis in LAL and LIF groups; **C**, OPLS-DA analysis in SAL and SIF groups; **D**, OPLS-DA analysis in LAL and LIF groups; **E**, Euclidean distance hierarchical clustering analysis visualizing the different intensity levels of characteristic metabolites in SAL and SIF groups; **F**, Euclidean distance hierarchical clustering analysis visualizing the different intensity levels of characteristic metabolites in LAL

and LIF groups. PCA: Principal component analysis; OPLS-DA: Orthogonal partial least-squares-discriminant analysis.

Supplementary Materials Table S1. The daily intake of DSS (g/day)

	ALD		IFD	
	Cage 1 (*n*=4)	Cage 2 (*n*=4)	Cage 3 (*n*=4)	Cage 4 (*n*=4)
Day 1	14.30	16.22	16.45	16.25
Day 2	16.29	16.75	16.36	18.04
Day 3	15.81	15.01	14.84	16.44
Day 4	13.06	14.44	14.13	15.94
Day 5	10.89	11.86	11.83	13.16
Day 6	9.78	10.73	11.95	9.86
Day 7	15.96	14.82	14.28	14.78

ALD: ad libitum-fed group with DSS; IFD: intermittent fasting group with DSS. SAL: Short-term of ad libitum-fed group; SIF: Short-term of intermittent fasting group; LAL: Long-term of ad libitum-fed group; LIF: Long-term of intermittent fasting group.
